# Region specific changes in nonapeptide levels during client fish interactions with allopatric and sympatric cleaner fish

**DOI:** 10.1371/journal.pone.0180290

**Published:** 2017-07-06

**Authors:** Marta C. Soares, Sónia C. Cardoso, Renata Mazzei, Gonçalo I. André, Marta Morais, Magdalena Gozdowska, Hanna Kalamarz-Kubiak, Ewa Kulczykowska

**Affiliations:** 1 CIBIO, Centro de Investigação em Biodiversidade e Recursos Genéticos, Universidade do Porto, Campus Agrário de Vairão, Vairão, Portugal; 2 Université de Neuchâtel, Institut de Biologie, Eco-Ethologie, Rue Emilie-Argand 11, Neuchâtel, Switzerland; 3 Genetics and Marine Biotechnology Department, Institute of Oceanology of the Polish Academy of Sciences, Sopot, Poland; Centre National de la Recherche Scientifique, FRANCE

## Abstract

Social relationships are crucially dependent on individual ability to learn and remember ecologically relevant cues. However, the way animals recognize cues before engaging in any social interaction and how their response is regulated by brain neuromodulators remains unclear. We examined the putative involvement of arginine vasotocin (AVT) and isotocin (IT), acting at different brain regions, during fish decision-making in the context of cooperation, by trying to identify how fish distinguish and recognize the value of other social partners or species. We hypothesized that the behavioural responses of cleaner fish clients to different social contexts would be underlain by changes in brain AVT and IT levels. We have found that changes in AVT at the level of forebrain and optic tectum are linked with a response to allopatric cleaners (novel or unfamiliar stimuli) while those at cerebellum are associated with the willingness to be cleaned (in response to sympatric cleaners). On the other hand, higher brain IT levels that were solely found in the diencephalon, also in response to allopatric cleaners. Our results are the first to implicate these nonapeptides, AVT in particular, in the assessment of social cues which enable fish to engage in mutualistic activities.

## Introduction

Social relationships are crucially dependent on individual ability to learn and remember beneficial cues [1]. The ability to pay attention to specific key information linked to other individuals or species is a prerequisite for animals that live in complex social systems. For instance, cues indicating that an animal is being watched, i.e., has an audience, lead to increases in levels of cooperation [2,3]. Social discrimination acquires a new dimension when occurring in cooperative or mutualistic contexts, as individuals’ decisions will determine the amount of benefits they gain but also depend on the responses of other individuals [4–6]. Typically, the term cooperation refers to a relationship between relatives in which both may asymmetrically benefit from the association [7,8], while the term mutualism is more specific to a similar cooperative relationship but this time, occurring between two different species [9].

Before engaging in any social interaction, animals need to assess the situation either by recognition of signalling cues associated with deceptive partners (e.g. individuals with mimicry strategies that exhibit specific shapes, colours or stripes [10]) or recollection of past interactions with less cooperative partners (e.g. partner that did not reciprocate equally in the past [11]).

Understanding how animals use new and previously acquired information to make decisions is particularly important to gain a clear view on: i) what makes individuals more, or less predisposed to cooperate, ii) how they respond to mixed cue signals (e.g. contradictory signals) that may differ in value and iii) how their responses are linked with brain neuromodulators. Among signalling neuromodulators are mammalian nonapeptides arginine vasopressin (AVP) and oxytocin (OT) and their teleostean homologs, arginine vasotocin (AVT) and isotocin (IT). They belong to an ancient family of neurohormones with an evolutionarily conserved structure and core physiological and behavioural functions across vertebrate taxa, particularly those related to regulation of vertebrate social behaviour [12,13,14]. In fish, the AVT/IT neurosecretory system contains three main cell groups distributed in the preoptic area (POA): gigantocellular, magnocellular and parvocellular, that project fibers to multiple target areas, such as ventral telencephalon, diencephalon, and various mesencephalic structures, in addition to projections to the neurohypophysis [15]. There is much evidence in fish that these neurohormones are implicated in aggressive behaviour [16,17], the promotion of territorial behaviour [18,19], changes in courtship behaviour [20,21], social status [22], pair formation [23], paternal care [24] and mutualistic behaviour [25–29].

One of the most well-known examples of cooperation between different species is the case of the interactions between cleaner fish and their client fishes. Cleaner fishes are specialized in inspecting the body surface, gill chambers and mouth of cooperating larger fishes, which are known as clients, in search of ectoparasites, mucus and dead or diseased tissues [30–34]. These cleaners must interact with a myriad of visiting species (dangerous piscivores to harmless herbivores) that may harbour more or less parasites and/or mucus, hence varying in value [35]. It is the quality of cleaners’ service that will determine the frequency of clients’ visits to their territories [see 36]. Regarding the best-known species of Indo-Pacific cleaner fish, *Labroides dimidiatus* there is emerging evidence pointing towards a crucial role of AVT as a modulator of their cleaning activities: intramuscular administration of AVT reduced their propensity to engage in interspecific cleaning activities and increased their motivation to engage with conspecific partners [25]. More recently, Cardoso and colleagues found that intramuscular AVT reduced a dimension of cooperativeness of individuals [26] and mediated associative learning abilities of cleaners depending on tasks (cue vs place discrimination) [27].

On the other side of these fish mutualisms, the clients, which regularly visit cleaners at their territories, face a different challenge: they must choose to visit amongst potential cleaner fish partners that may cooperate (e.g. that mostly forage on client’s ectoparasites), or not (e.g. cleaners that also feed on clients’ mucus which is detrimental to clients’ organism; for review see [36]). During their early life stages clients need to learn to seek, recognize and interact with several cleaners, identifying signaling cues such as their specific colors, stripes or shape to get the best service, i.e. removing the ectoparasites or gaining physical stimulation. Recent research has demonstrated that clients that interact more frequently with cleaners have better body condition [37] and the recurrent physical contacts contribute to reducing their stress levels [38]. The level of benefit arising from these interactions depends on ability of client fish to identify the specific cues to safely approach the fish providing an honest, fair cleaning service and avoid dubious cues, such as those coming from “false” or mimic cleaners. The false cleaners copy both appearance and behaviour of obligatory cleaners as *L*. *dimidiatus* but instead of cleaning they bite their clients [39]. However, the mechanisms involved in the regulation of such behaviour has not yet been determined, particularly those that help naïve coral reef fishes to learn and remember novel information, which they can use in future interactions.

There are only few studies that measured AVT and IT concentrations in different regions of fish brain in order to link them with the expression of different social behaviours [see 40,41], and only two that focus specifically on mutualistic behaviour [28,29]. In our previous study on AVT levels in different brain regions in four species of Labrid fish, we found that in the cerebellum of the obligate cleaners *L*. *dimidiatus* and *Labroides bicolor*, the levels were higher than those in facultative cleaner species and a non-cleaner species [29]. We suggested that AVT levels in the cerebellum can be associated with the expression of mutualistic behaviour. Furthermore, higher levels of AVT in the whole brain and forebrain of the obligate *L*. *bicolor* were associated with an increase of aggressiveness towards clients and roaming behaviour [29]. In another study, Cardoso and colleagues identified a link between brain isotocin levels and the quality of relationship within mixed sex couples of cleaners, i.e. forebrain IT levels were higher in those males that received more tactile stimulation from female partners [28]. Therefore, further research on the distribution of AVT and IT across different brain areas, in which they are hypothesized to act, is a promising approach.

**Fig 1:**
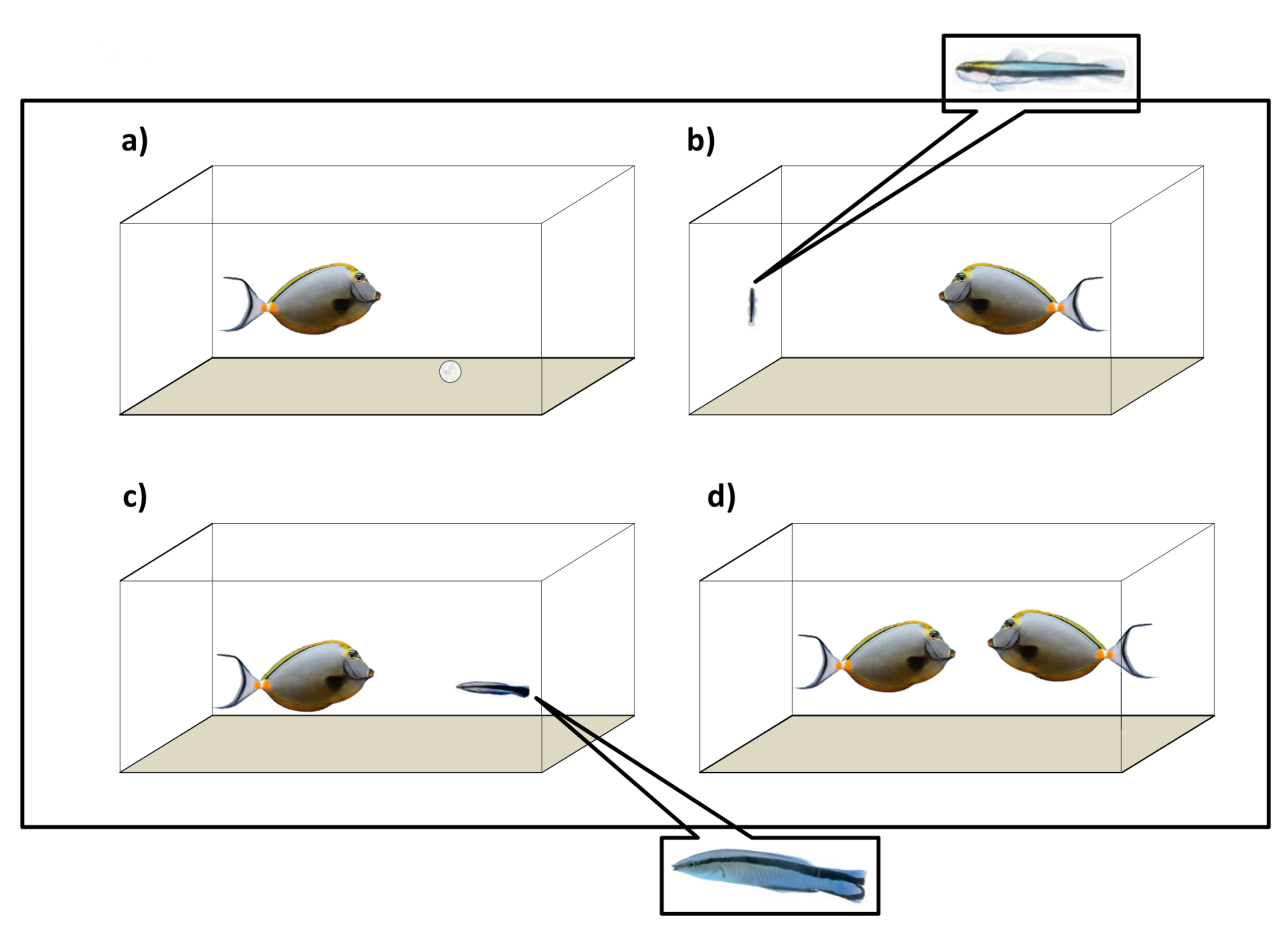
Experimental setup. (A) Individual client fish (Indo-Pacific *Naso elegans*) is introduced to a novel object (a white ball), (B) Individual client fish is introduced to an allopatric cleaner species (the Caribbean cleaning goby *Elacatinus evelynae*), (C) Individual client fish is introduced to a sympatric cleaner species (the Indo-Pacific cleaner wrasse *Labroides dimidiatus*) and (D) Individual client fish is introduced to a conspecific. Images of fish are not in real scale, particularly those of the cleaner fish *L*. *dimidiatus* and *E*. *evelynae* when compared with client *N*. *elegans* size.

Here, we investigate if different signals are translated into changes in behaviour and brain nonapeptide’ levels in a coral reef fish, the Indo-Pacific blonde naso tang *Naso elegans* (family Acanthuridae). Naso tang is a potentially frequent client of the cleaner wrasse *L*. *dimidiatus* (both species originate from the Indo-Pacific region) and can be easily adapted to laboratory conditions. The aim of the present study is to examine the role of AVT and IT, acting at different brain regions, in client behavioural responses to cleaners. In laboratory experiments, we introduced wild-caught clients (Indo-Pacific *N*. *elegans*) to different social partners (experimental treatments) using a sympatric or recognizable (Indo-Pacific conspecifics and heterospecifics) and allopatric or non-recognizable (Atlantic originated heterospecifics, see Fig 1) cleaners. We assumed that the wild-caught clients encountered sympatric cleaner species before in their habitat as they are ubiquitous and occupy the same areas. We hypothesized that clients’ behavioural response to different social partners (experimental treatments) would be associated with changes in brain AVT and IT levels.

## Materials and methods

### Ethical note

The protocols were carried out in accordance to the approved guidelines by the Oceanário de Lisboa (fish housing facilities), where the experiments were then developed. Animal procedures used in this study were also approved by the Portuguese Veterinary Office (Direcção Geral de Veterinária, license # 0420/000/000/2009).

### Animals and housing

Experiments were conducted at the fish housing facilities of the Oceanário de Lisboa (Lisbon, Portugal). The specimens used in this study were adult blonde naso tang *Naso elegans* (family Acanthuridae, also known as clients), the Indo-Pacific bluestreak cleaner wrasse *Labroides dimidiatus* and the Caribbean sharknose cleaning goby *Elacatinus evelynae*), all imported to Portugal by a local distributor (Tropical Marine Centre, Lisbon, Portugal). Total length (TL) and total weight (TW) of tang *N*. *elegans* ranged from 6.9 to 15.5 cm (mean ± SE: 10.41 ± 0.34 cm) and 4.9 to 78.1 g (19.66 ± 2.15 g). Tangs were kept in stock aquaria of 100x40x40 cm and cleaning gobies in aquaria of 50x40x40 cm, in groups of 5 to 10 individuals, while cleaner wrasses were kept alone in 50x40x40 cm aquaria. All aquaria were combined in a flow through system that pumped water from a larger sump (150x50x40 cm) that served as a mechanical and biological filter. Nitrite concentration was kept to very low levels (always below 0.3 mg/L). Each tank contained an air supply and a commercial aquarium heater (125W, Eheim, Jäger). PVC pipes (15–20 cm long; 20 cm diameter) served as shelter for the fish. Experiments were carried out between September and November 2012 in the individual smaller tanks (50x40x40 cm).

### Experimental design and sampling

As mentioned previously, because the clients *N*. *elegans* and the cleaner wrasse *L*. *dimidiatus* both originated from Maldives (Indian Ocean), the latter were classified as sympatric cleaners in contrast to the Caribbean (Atlantic Ocean/Caribbean) originated cleaning gobies classified as allopatric cleaners. On each test day, the following experimental treatment groups were randomly allocated to focal clients (subjects): a) ball (a heavy, white ball, about 5 cm in diameter, permanently attached to the bottom), b) sympatric cleaner (*L*. *dimidiatus*), c) allopatric cleaner (*E*. *evelynae*) and d) conspecific (*N*. *elegans*), see Fig 1. Clients were distributed across experimental treatments as follows: 11 individuals for the sympatric cleaner group, 8 for the allopatric cleaner group, 10 for the conspecific group and 11 for the ball group. Each client was introduced into the experimental tank and left for at least 2–5 min until regular activity was restored (i.e. individuals were swimming normally). Experimental aquaria were also divided by opaque partitions preventing any visual contact between fish during experiments. Behaviour was then videotaped for the next 60 minutes while the experimenter left the room (see behavioural analyses section below). At the end of experiments, each tang was rapidly captured and sacrificed with an overdose of tricaine solution, a powerful anesthetic (MS222, Pharmaq; 500–1000 mg/L) and the spinal cord sectioned (both methods aimed to reduce fish suffering). The brain was immediately dissected under a stereoscope (Zeiss; Stemi 2000) into five macro-areas: forebrain (olfactory bulbs + telencephalon), diencephalon, optic tectum, cerebellum and brain stem, by separating along the natural boundaries between macro-areas, using the same methodology applied by [40,41,42] (see the dissection procedure at S1 File). Major brain areas were weighed and stored at—80°C.

### Quantification of nonapeptides by high performance liquid chromatography with fluorescence detection (HPLC-FL)

Brain samples weights were used for later calculation of nonapeptide’ levels (peptide content was expressed per milligram of brain sample). Then, they were sonicated in 1 mL Milli-Q water (MicrosonXL, Misonix, USA), acidified with glacial acetic acid (3 μL) and placed in a boiling water bath for 3.5 min. Next, homogenates were centrifuged (12,000 *g*, 20 min, 4°C) and supernatants decanted and loaded onto previously conditioned (1 mL methanol, 1 mL Milli-Q water) solid phase extraction (SPE) columns (30 mg/1 mL, Strata-X, Phenomenex). To purify samples, columns were washed successively with 0.6 mL Milli-Q water and 0.6 of mL of 0.1% TFA (trifluoroacetic acid) in 5% acetonitrile. The peptides were eluted using 1.2 mL of 80% acetonitrile. The eluate was evaporated to dryness using a Turbo Vap LV Evaporator (Caliper Life Scence, USA). Samples were then frozen and stored at -80^°^C prior to HPLC analysis.

Before quantitative analysis, the samples were re-dissolved in 40 μL of 0.1% TFA in 30% acetonitrile and divided into two for replication. Pre-column derivatization of AVT and IT was performed according to the procedure by Gozdowska and colleagues [43]. For derivatization reaction, 20 μL of sample and 20 μL of 0.2 M phosphate buffer (pH 9) were mixed, and then 3 μL of NBD-F (4-fluoro-7-nitro-2,1,3-benzoxadiazole: 30 mg in 1 mL of acetonitrile) was added. The solution was heated at 60^°^C for 3 min, cooled on ice, acidified with 4 μL of 1 M HCl and eluted in a HPLC column. Derivatized samples were measured with Agilent 1200 Series Quaternary HPLC System (Agilent Technologies, USA). Chromatographic separation was achieved on an Agilent ZORBAX Eclipse XDB-C18 column (150 mm x 4.6 mm I.D., 5 μm particle size). The gradient elution system was applied for separation of derivatized peptides. The mobile phase consisted of solvent A (0.1% TFA in H_2_O) and solvent B (0.1% TFA in acetonitrile: H_2_O (3:1). A linear gradient was 40–65% of eluent B in 20 min. Flow rate was set at 1 mL/min and the column temperature set to 20 ^o^C. Injection volume was 47 μL. Fluorescence detection was carried out at 530 nm with excitation at 470 nm (calibration data and curves are provided at Table A and Fig A in S1 File).

### Behavioural analyses

During each video analysis, we recorded for the focal client *N*. *elegans* the following measures, during the 60 minutes of observation: 1) the number and duration (in seconds) of each cleaning inspection received; 2) the frequency and duration of tactile stimulation received (where a cleaner touches, with ventral body and fins, the body of the client and no feeding is involved [44]); 3) the number of jolts by clients (cleaners sometimes bite clients and they respond with a short body jolt which usually is a behaviour associated with cheating by cleaner fish [45,46]); 4) number and duration of chases that occurred when the focal individual rapidly advanced toward the other (either a cleaner or conspecific, in seconds) and finally 5) number of bites provided by focal individual. Moreover, it is important to note that the context in which these chases occur (either against sympatric cleaners or against other conspecifics) may be quite distinct. In the wild, this usually happens that clients chase cleaners, because cleaners were aggressive towards the clients or clients jolted previously [47]. In the conspecific context, the incidences of chases by the subject may be due to size differences (if intruder is larger than the resident) or to sex differences. Although we tried to match the sizes of the individuals, this was not always possible.

### Statistical analyses

A total of 40 clients were used for brain AVT and IT measurement. Of these, according to E_2_ measurements (see S1 File for protocol), 18 were males (mean ± SD: 0.0045 ± 0.0022 ng/mg) and 22 were females (0.057 ± 0.073 ng/mg). In four individuals, AVT levels were below the limit of detection. Brain IT and AVT levels (brain and behavioural variables) were log transformed to conform to parametric parameters of homogeneity of variances (assessed by Levene’s test). In each experimental context the following behavioural measures were calculated: a) the frequency of cleaning interactions (per 60 min), b) mean inspection duration (in seconds), c) proportion of interactions in which tactile stimulation was applied to clients (number of interactions in which tactile stimulation was provided/ total number of interactions), d) frequency of jolts per 100s of inspection, e) frequency of antagonistic charges (chases per 60 min), f) duration of chases (in seconds) and g) frequency of bites given by focal individual (per 60 minutes). Brain levels of AVT and IT for the whole brain and each brain macro-area (forebrain, diencephalon, optic tectum, cerebellum and brain stem) were first compared by using two way independent measures, with sex and treatment as fixed factors. Because sex was not found to be a significant factor, it was the dropped from all models: forebrain (AVT): F_(3,35)_ = 0.191, p = 0.902; forebrain (IT): F_(3,36)_ = 1.796, p = 0.168; diencephalon (AVT): F_(3,35)_ = 0.126, p = 0.944; diencephalon (IT): F_(3,36)_ = 0.774, p = 0.517; optic tectum (AVT): F_(3,35)_ = 1.051, p = 0.384; optic tectum (IT): F_(3,36)_ = 1.816, p = 0.160; cerebellum (AVT): F_(3,35)_ = 0.653, p = 0.588; cerebellum (IT): F_(3,36)_ = 0.279, p = 0.840; brain stem (AVT): F_(3,35)_ = 0.866, p = 0.156; brain stem (IT): F_(3,36)_ = 0.109, p = 0.955). One way ANOVAs, followed by Tukey post-hoc HSD tests tested for the effect of treatment on AVT and IT brain levels. Relationships within and between behavioural measures and clients’ brain neuropeptide levels were examined by using the Pearson correlation coefficients. The Hochberg-adjusted p-values [48], which provide adjusted alpha values to account for multiple comparisons, were calculated in order to account for multiple comparisons. Therefore, we report the exact p-value produced for each correlation analysis and indicate whether it fell below its adjusted alpha. All tests were two tailed, the ANOVAs were performed with the R software [49] and Pearson correlations were performed in the software package SPSS Statistics, version 22.

## Results

### Client behaviour

There were differences in the behavioural response of client *Naso tang* (hereafter referred to as clients) across experimental treatments (Fig 1). Behavioural interactions occurred in two experimental treatments: clients interacted with the sympatric cleaner fish (*L*. *dimidiatus*) as well as with conspecifics (see Table 1). Clients that were cleaned more frequently by sympatric cleaners appeared to receive less tactile stimulation (Pearson correlation test: r = - 0.64, N = 10, P = 0.04), were observed to jolt more frequently (r = 0.66, N = 10, P = 0.04) and also chased cleaners more often (r = 0.72, N = 10, P = 0.02). The frequency of jolts during inspection was significantly correlated with the frequency of chases (r = 0.88, N = 10, P = 0.001). When introduced to an allopatric cleaner species (cleaning gobies), clients avoided contact with gobies and did not approach the gobies at all. Clients did not approach or have any contact with the ball either (see Table 1).

**Table 1:** Frequency of observed behavioural measures for each experimental treatment. These include: a) the frequency of cleaning interactions, b) mean inspection duration, c) proportion of interactions in which tactile stimulation was applied to clients, d) frequency of jolts per 100 s of inspection, e) frequency of chases, f) duration of chases and g) frequency of bites given by focal individual. Mean ± Standard Error (SEM) are provided for each behavioural measure.

Behaviour	Experimental treatments			
	Sympatric cleaner	Allopatric cleaner	Conspecific	Ball
Frequency of cleaning interactions	14.00 ± 2.75	0	0	0
Inspection duration (in sec)	7.33 ± 1.22	0	0	0
Proportion of interactions with tactile stimulation	0.17 ± 0.04	0	0	0
Proportion of time providing tactile stimulation	0.27 ± 0.06	0	0	0
Jolts (per 100s of inspection)	0.42 ± 0.22	0	0	0
Frequency of chases (by focal)	0.90± 0.54	0	6.00 ± 2.85	0
Chase duration (in sec)	1.20± 0.51	0	22.00 ± 10. 44	0
Frequency of bites (provided by focal)	0	0	0.45 ± 0.33	0

### Client brain neuropeptides

**Fig 2:**
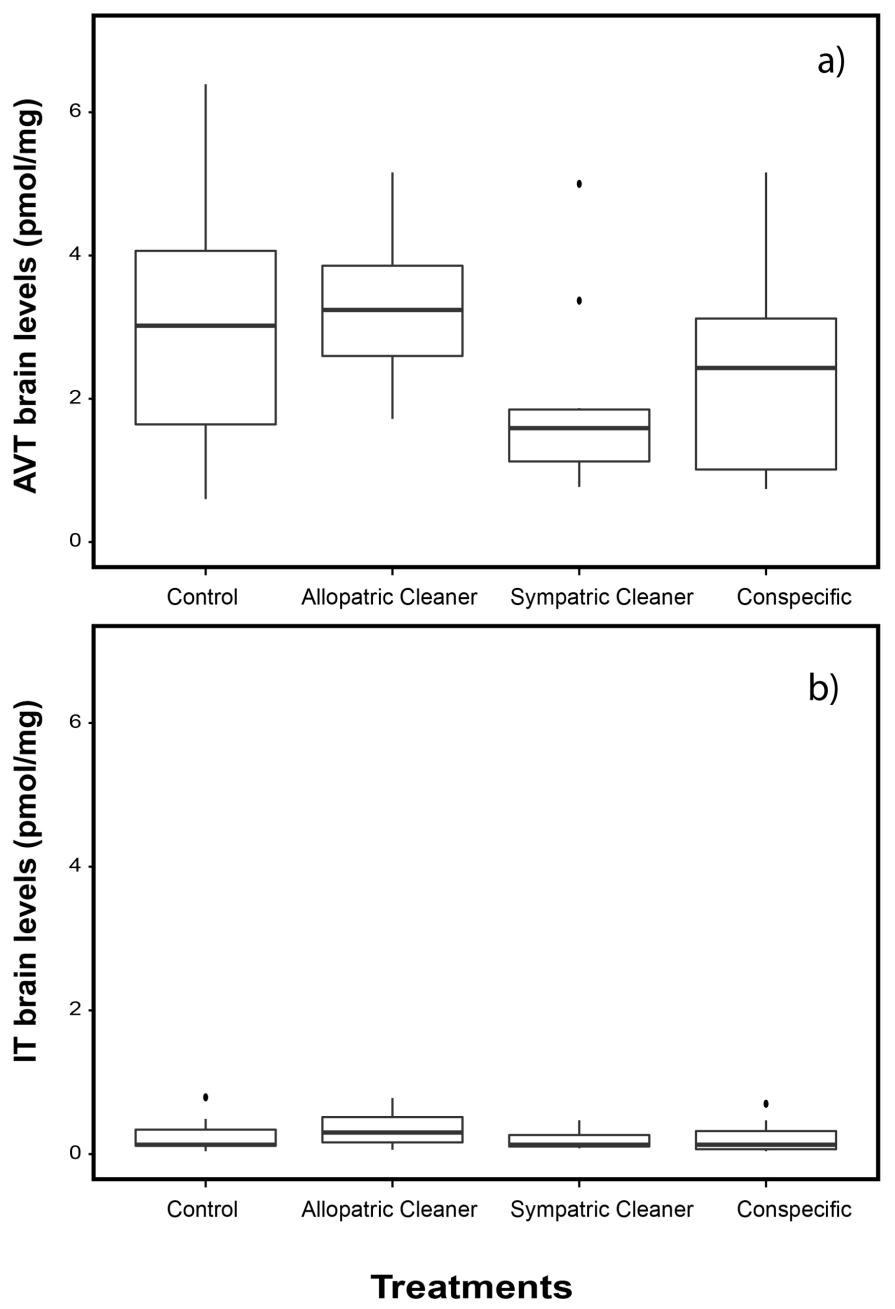
Whole brain nonapeptide’ levels in clients (*Naso elegans*). (A) levels of arginine vasotocin (AVT) and (B) levels of isotocin (IT) in four treatment groups: a) sympatric cleaner (*Labroides dimidiatus*), b) allopatric cleaner (*Elacatinus evelynae*), c) conspecific (*N*. *elegans*) and d) ball, expressed as AVT (pmol/mg) and IT (pmol/mg). Medians (full lines) and interquartile range are presented in boxes.

**Fig 3:**
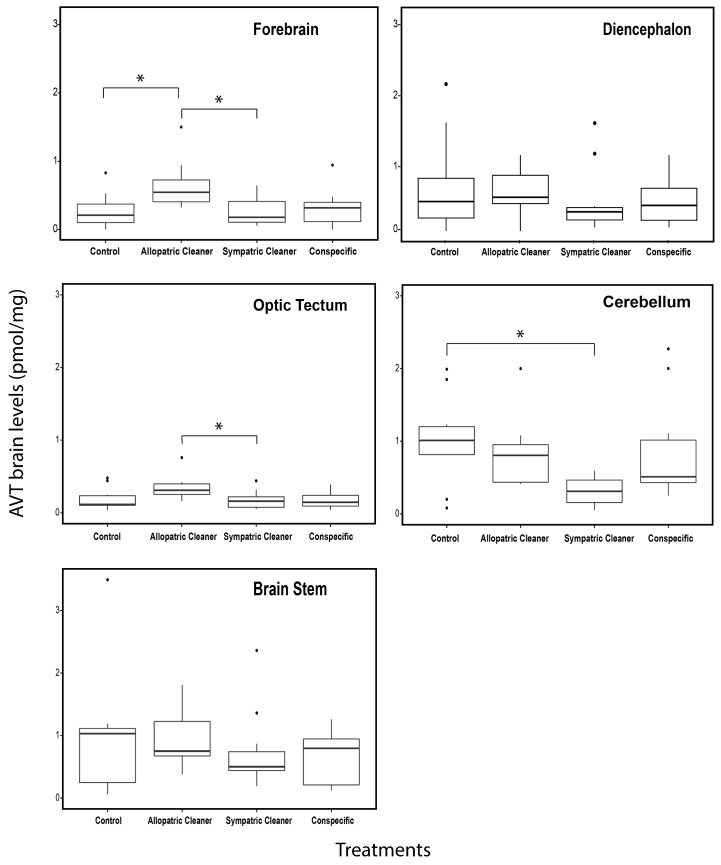
Levels of arginine vasotocin (AVT) in different brain macro-areas: Forebrain, diencephalon, optic tectum, cerebellum and brain stem in clients (*Naso elegans*). Four treatment groups: a) sympatric cleaner (*Labroides dimidiatus*), b) allopatric cleaner (*Elacatinus evelynae*), c) conspecific (*N*. *elegans*) and d) ball, expressed as AVT (pmol/mg). Medians (full lines) and interquartile range are presented in boxes.

**Fig 4:**
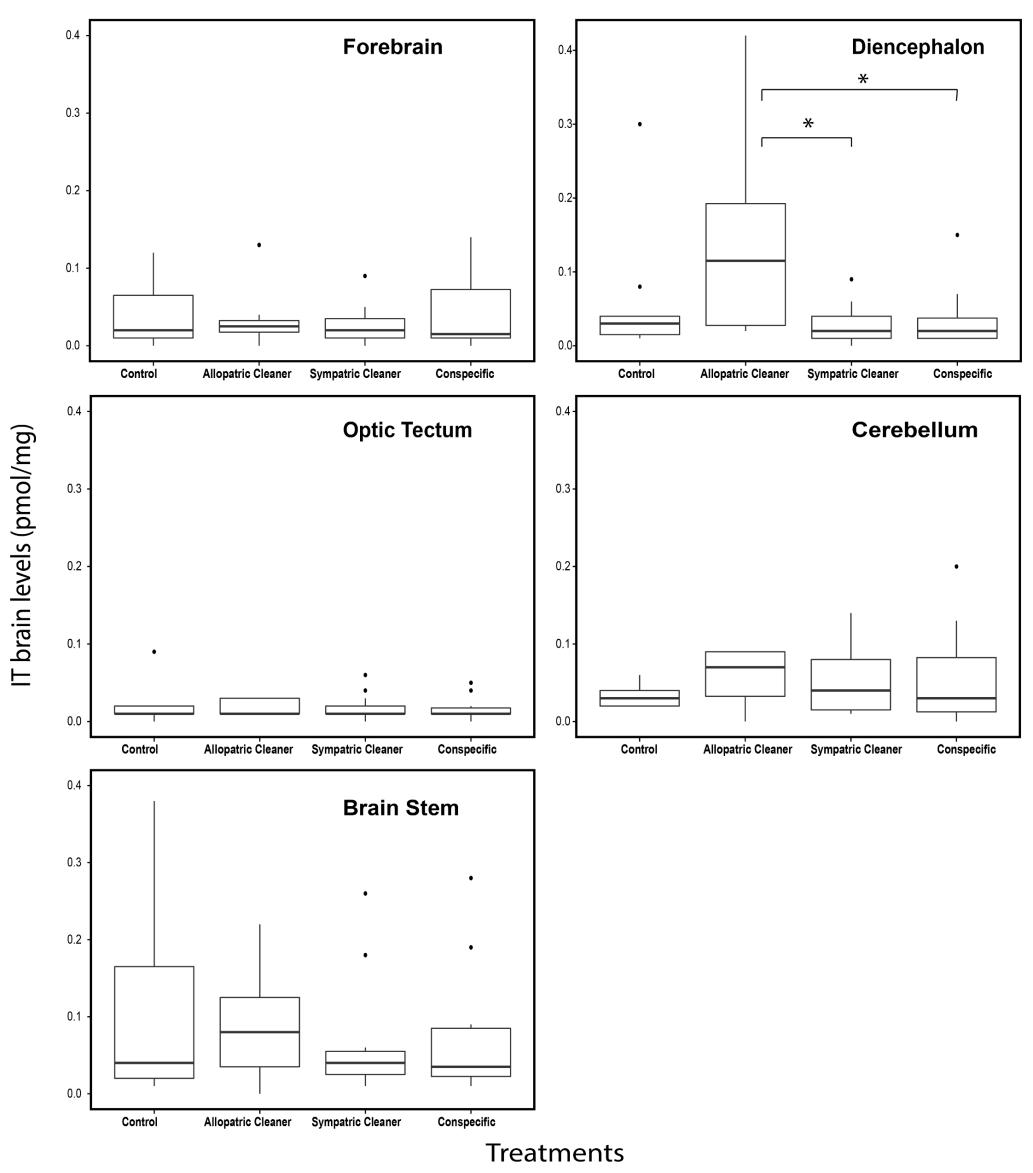
Levels of isotocin (IT) in different brain macro-areas: Forebrain, diencephalon, optic tectum, cerebellum and brain stem in clients (*Naso elegans*). Four treatment groups: a) sympatric cleaner (*Labroides dimidiatus*), b) allopatric cleaner (*Elacatinus evelynae*), c) conspecific (*N*. *elegans*) and d) ball, expressed as IT (pmol/mg). Medians (full lines) and interquartile range are presented in boxes.

Mean concentrations of AVT and IT in the whole brain and brain macro-areas are given in Figs 2, 3 and 4 and Table B in S1 File. No differences were found in whole brain AVT levels across experimental treatments (one way ANOVA, F_3,36_ = 2.183, *p* = 0.107, Fig 2A).

Brain AVT levels differed significantly at the forebrain, optic tectum and cerebellum across experimental treatments (forebrain: F_3,35_ = 3.282, *p* = 0.032; optic tectum: F_3,35_ = 3.274, *p* = 0.032 and cerebellum: F_3,35_ = 4.822, *p* = 0.006, see Fig 3, Table B in S1 File) but not at the diencephalon or brain stem (diencephalon: F_3,35_ = 0.365, *p* = 0.779 and brain stem: F_3,35_ = 0.628, *p* = 0.602, see Fig 3, Table B in the S1 File). Higher brain AVT levels were found at the forebrain and optic tectum of clients in contact with allopatric cleaners when compared with those introduced to sympatric cleaners (forebrain: p = 0.03; optic tectum: p = 0.04, see Fig 3, Table C in S1 File); and at the forebrain of those in contact with allopatric cleaners compared to those with a ball (forebrain: p = 0.035, Fig 3, Table C in S1 File). Clients in contact with sympatric cleaners showed significantly lower levels of brain AVT in the cerebellum, when compared to those introduced to a ball (p = 0.01, Fig 3, Table C in S1 File), while no significant differences were found when clients were in direct contact with conspecifics and allopatric cleaners (client introduced to sympatric cleaner vs conspecific p = 0.07; client introduced to sympatric vs allopatric cleaner p = 0.07, Fig 3, Table C in S1 File).

No differences were found in whole brain IT levels across experimental treatments (one way ANOVA, F_3,36_ = 0.896, *p* = 0.452, Fig 2B). Brain IT level differed significantly only at the diencephalon (diencephalon: F_3,36_ = 3.8, *p* = 0.02; Tables B and D in S1 File, for results in other brain macro-areas, Fig 4). Clients in contact with allopatric cleaners had higher brain IT level at the diencephalon compared with those introduced to sympatric cleaners or conspecifics (clients introduced to allopatric vs sympatric cleaners, p = 0.03; and allopatric cleaners’ vs conspecifics, p = 0.03, see Table D in S1 File, Fig 4).

Regarding the relationship between client behaviour and brain neuropeptide, none of the correlations remained significant after calculation of Hochberg-adjusted p-values [48] (Tables E and F in S1 File).

## Discussion

In nature, individuals must navigate complex social environments. Here, we introduced clients to treatments that differed substantially: a) the conspecific context was both familiar and social, b) the sympatric cleaner condition was familiar, cooperative, and social, c) the allopatric cleaner condition was novel, social, and potentially unsafe and d) the control condition was non-social and novel. Specifically, we showed that clients i) engaged mostly in agonistic interactions with conspecifics, primarily on cleaning interactions with sympatric cleaners, ii) did not engage in agonist or cleaning interactions with allopatric cleaners and iii) were similarly interactively absent in the control context (see Fig 1 for experimental setup); we found that both nonapeptide levels (AVT and IT) differed overall across some specific brain regions, changing in accordance to specific treatment. We found that clients in contact with allopatric cleaners had higher levels of AVT in the forebrain and the optic tectum than those introduced to sympatric cleaners. On the other hand, clients in contact with sympatric cleaners showed lower levels of AVT in the cerebellum compared to those kept with a ball. Moreover, levels of IT were higher in subjects in contact with allopatric cleaners when compared to sympatric cleaners and conspecifics only at the diencephalon. Our results are the first to link these nonapeptides with the variation of client social behaviour and probability to engage in cooperative interactions in fish.

Clients in contact with never-before seen cleaning gobies, a species of Atlantic (Caribbean) origin, shared a common behavioural response: they kept at a distance and never interacted. Some may have ventured and explored, e.g. got to observe the gobies a bit closer, but only temporarily. Nonetheless, clients seemed to be extremely aware of goby’ presence (for instance, by moving when gobies moved sharply in their direction), perhaps in trying to determine the potential risk associated to these cleaning gobies. These cleaning gobies share common features with other cleaner fish species, such as contrasting colorful stripes, which they use to advertise their cleaning services [50,51]. For example, the Caribbean cleaning gobies *E*. *evelynae* (used in this study) also display specific phenotypic features such as blue and yellow stripes, which normally signal cleaning service for coral reef fish [51] and evokes clients’ curiosity. However, the display of conspicuous color stripes is also used by other species to signal other forms of communication, which is the case of some cleaner fish mimics [52,53]. Considering the lack of response by clients introduced to a novel object (ball), especially when compared with those introduced to a novel cleanerfish species (gobies), novelty was probably not the sole factor influencing these animals’ response to gobies.

We found that subject clients, introduced to an allopatric cleaner, had higher concentrations of forebrain AVT levels compared to those in contact with a sympatric cleaner fish species and those presented to a static white ball. A similar result was observed at the optic tectum (an area of visual integration), with clients having higher AVT levels when in contact with allopatric cleaners compared to sympatric cleaners. In teleost fish, the forebrain, namely the dorsomedial (Dm) telencephalon, a partial homolog region of the mammalian amygdala, is known to be involved in fear based conditioning (learning to avoid noxious or harmful stimuli) [54–57]. Mapping of AVT receptors in the brain of teleost species have shown that V1a receptors are widely distributed throughout the forebrain, being specifically present on the Dm region but also at optic tectum [58–61]. Thus, the increase of AVT levels in both the optic tectum and the forebrain regions of those in contact with allopatric cleaners (compared to sympatric cleaners and control) provides an indication that AVT is conveyed to these areas and may be involved in the perception of novel and potential noxious /unsafe stimuli.

Clients in contact with a sympatric cleaner species had lower AVT levels at the cerebellum when compared with those introduced to a ball (control). Interestingly, when looking at the initial correlation trends, before correcting the p values, there seemed to be a negative link between the duration of clients’ interaction with native cleaners and a reduction of AVT levels in the cerebellum. Hence, it appears that one of the most likely reasons for AVT decrease at the cerebellum is the length of interactions with cleaners. These results are in line with previous studies by Soares and colleagues, done in the wild, in which exogenously infused AVT decreased the likelihood of engaging of native cleaners (*L*. *dimidiatus*) in cleaning interactions with their clients [25]. Thus, lower levels of AVT at the cerebellum may be linked to a higher propensity to interact with cleaners. Furthermore, the cerebellum of teleost fish, which is traditionally associated with motor control, is also highly implicated in several cognitive and emotional functions, particularly in classical conditioned /associative learning and memory processes [62,63]. Indeed, it is through classical associative learning that clients first acquire the iterated training that makes them visit these cleaners’ territories, by associating the interaction with a cleaner with benefits, such as: lowering parasite levels, gaining tactile stimulation, or cortisol levels reduction [64]. Thus, there is a potential higher scope for the influence of AVT on cleaners’ learning abilities happening at the cerebellum level but most likely in straight connection with other important brain areas, such as the telencephalon. However, because we do not have any direct proof that each of the subjects used had already been in contact with cleaners (before experimental treatments), we cannot be sure that the effect observed in AVT levels is due to learned memory/recognition or to genetically-inherited predisposition to interact with cleaner species. In future studies would be interesting to include a previous manipulation of individual subject familiarity in relation to each cleaner and/or conspecific introduced.

In the present study, higher values of IT were found solely at the diencephalon, in subject clients put in contact with allopatric cleaning gobies compared to those interacting with sympatric cleaner fish species or conspecifics. Recent studies have shown that intranasal administration of OT given in different social situations, is able to enhance the salience of social cues, especially when it is associated with the activation of the dopaminergic system (for review see [65]). For instance, in humans, OT activates Ventral Tegmental Area (VTA) in response to social relevant cues signaling reward or punishment, e.g. friendly or angry faces [66]. Similar studies have also been done in rodents: for example, administration of OT antagonist during mating of individuals that previously acquired strong partner preference seems to reduce their motivation to seek further contact [67]. In teleost fish, the posterior tuberculum (PT) located in the basal diencephalon is the most likely homolog region of mammalian VTA [68]. Perhaps, the occurrence of higher levels of IT at the diencephalon could be a response to the novelty associated to the allopatric cleaners; whether it enhances the dopaminergic activity at the PT remains to be studied.

In conclusion, it appears that in response to unclear/novel and potentially noxious stimuli, nonapeptide’ influence is reported at relevant brain areas: AVT at the level of the optic tectum and the telencephalon (forebrain), and IT at the level of the diencephalon. Moreover, in response to sympatric cleaners, clients showed reduced levels of AVT in the cerebellum, however no differences were found in other areas, between contexts. It is perhaps the overall AVT expression across brain areas (reduction at the cerebellum but maintenance at the other areas) that works as a pre-requisite for mutualistic behaviour to occur. Future complementary research should further test for the direct influence of exogenous administration of AVT and IT on client ability to recognize and interact with cleaners (direct comparison between sympatric and allopatric cleaners). Moreover, it would be worthwhile to extend these tests to juvenile naïve clients, to find out if these would respond similarly to sympatric cleaners as the adult clients do in relation to allopatric cleaners.

## Supporting information

S1 File In this file are included Tables A, B, C, D, E, F, Fig A. and two methodological descriptions: 1) Subject clients’ brain microdissection procedure and 2) Measurement of gonad 17β-estradiol (E2) for sex identification.
(DOCX)
