# Supplementary material for: Region specific changes in nonapeptide levels during client fish interactions with allopatric and sympatric cleaner fish
Source: PLoS One. 2017 Jul 6;12(7):e0180290. doi: 10.1371/journal.pone.0180290 (PMC5500320; doi:10.1371/journal.pone.0180290)
Supplement: S1 File — In this file are included Tables A, B, C, D, E, F, Fig A. and two methodological descriptions: 1) Subject clients’ brain microdissection procedure and 2) Measurement of gonad 17β-estradiol (E2) for sex identification. (DOCX) [file pone.0180290.s001.docx]

**Supporting information**

Region specific changes in nonapeptide levels during client fish interactions with allopatric and sympatric cleaner fish

Marta C. Soares^1*^, Sónia C. Cardoso^1^, Renata Mazzei^1,2^, Gonçalo I. André^1^, Marta Morais^1^, Magdalena Gozdowska^3^, Hanna Kalamarz-Kubiak^3^, Ewa Kulczykowska^3^

1 CIBIO, Centro de Investigação em Biodiversidade e Recursos Genéticos, Universidade do Porto, Campus Agrário de Vairão, 4485-661 Vairão, Portugal

2 Université de Neuchâtel, Institut de Biologie, Eco-Ethologie, Rue Emilie-Argand 11, 2000 Neuchâtel, Switzerland

3 Genetics and Marine Biotechnology Department, Institute of Oceanology of the Polish Academy of Sciences, Sopot, Poland

**Author for correspondence:** Marta C. Soares

email: [marta.soares@cibio.up.pt](mailto:marta.soares@cibio.up.pt)

(1) Subject clients’ brain microdissection procedure

After removing the dorsal portion of the skull with the use of forceps, we started the dissection by removing the cerebellum. The cerebellum is dorsally located and connected with the brainstem. The cerebellum connection with the brain stem is anteriorly located and is comprise to a narrow area. We pulled up the cerebellum bulb and made a horizontal cut beneath it ensuring that all the cerebellum was collected. Without the cerebellum, we have access to the most anterior part of the brainstem. The brainstem is anteriorly connected with both diencephalon and optic tectum. We made a vertical cut separating the brainstem from the diencephalon and optic tectum. The cut location was determined by looking at the skull morphology as well as where the optic tectum bulbs rested. The cut was made one millimetre posteriorly to the end of the optic tectum and before the deepening of the skull structure. Next, we dissected the optic tectum, we started by lifting the optic tectum bulbs and looked for the sulk in the skull morphology, a morphological landmark marking the beginning of the diencephalon brain region. We also look for the presence of a highly myelinated region that marks the link between the diencephalon and the optic tectum (characterised by is whiter coloration). When separating the two macro areas, we included this highly myelinated region in the optic tectum. After cutting the connection between the optic tectum, the diencephalon and the optic nerve, we proceed to cut the connection that optic tectum hold with the telencephalon. Knowing that between both structures we have the preoptic brain region, we carefully pulled the optic tectum posteriorly and cut the connection as close as possible to the telencephalon bulbs. At this point, the diencephalon is just resting in the skull, so it is easily removed without losing any integral part of the brain region. The last macro area to be removed was the telencephalon (together with the olfactory bulbs). The olfactory bulbs on these species are small structures that are difficult to dissociate of the telencephalon. Carefully we pulled the telencephalon posteriorly and cut the remaining connections that these regions have with the skull structure.

All the dissections were done by the same researcher and the dissection protocol optimised using test individuals.

(2) Measurement of gonad 17β-estradiol (E_2_) for sex identification

The gonads were weighed for calculations of 17β-estradiol (E_2_) concentration as nanogram per gram of wet gonad. Gonads were individually sonicated in 0.5 mL of phosphate buffer (0.05 M, pH 7.4) supplemented with sodium azide (NaN_3_) using a Microson TM XL 2000 (USA). The sonicates were centrifuged at 20,000 *g* for 20 min at 4°C and the supernatants stored at - 70°C prior to the analysis of E_2_ levels. The E_2_ concentration in gonad organic extracts was determined using a Spectria Estradiol radioimmunoassay (RIA) kit (Orion Diagnostica, Finland). Gonad supernatants (200 mL) were extracted with 1.6 mL of ethyl ether according to the method in^1^ after modifications. Samples were vortexed for 1 min at 350 *g*, for 30 min at 35 g and then held at -20°C for 30 min to separate the layers. The ethyl ether layer was decanted into a glass tube and evaporated under a stream of nitrogen. Dried extracts were stored at -20°C prior to analysis. The recovery rate of the extraction was between 86–109%. Extracts were dissolved in phosphate buffer (0.05 M, pH 7.4) supplemented with NaN_3_ and samples of 100 mL were taken for RIA analysis. Iodinated E_2_ with ^125^I was used as a tracer. A standard curve was prepared using six standard dilutions of 50, 150, 500, 1500, 5000 and 15,000 pmol/L. The assay was conducted in RIA tubes according to the kit manufacturer’s instructions with slight modifications. The samples were added to tubes that had been pre-coated with polyclonal anti-rabbit antiserum. After vortexing for 10 seconds, the tubes were incubated for 2 hours at 37°C, decanted, washed with 1 mL of Tween 20 solution (6 x concentrated) and decanted again. Radioactivity in each tube was measured for 1 min using a Wallac Wizard 1470 gamma counter (Perkin Elmer Life Science, USA). All samples were assayed in duplicate. The detection limit of the assay was 37 pmol/L. The intra-assay coefficient of variation was 6.5%. The inter-assay variation was not determined because all samples were measured in the same assay.

**Table A.** Calibration data used for the quantification of nonapeptides (arginine vasotocin - AVT and isotocin - IT) by high performance liquid chromatography with fluorescence detection (HPLC-FL).

| No | **AVT** | | | **IT** | | |
| --- | --- | --- | --- | --- | --- | --- |
|  | Retention time (min) | Concentration  (pmol/mL) | Peak area | Retention time (min) | Concentration  (pmol/mL) | Peak area |
| 1 | 12.128 | 1040 | 357.65 | 15.059 | 1326 | 888.74 |
| 2 |  | 520 | 177.02 |  | 663 | 407.9 |
| 3 |  | 260 | 83.19 |  | 331.5 | 191.44 |
| 4 |  | 104 | 48.90 |  | 133 | 130.72 |
| 5 |  | 10.4 | 11.93 |  | 13.3 | 19.7 |

**Table B**. Arginine vasotocin (AVT) and isotocin (IT) levels (mean ± SEM; pmol mg^‐1^) in different brain macro-areas of four experimental treatments (examined with One Way ANOVAs). F-statistic (F), p-values (p) and adjusted R-squared values are presented.

|  |  | ***Experimental treatments*** | | | |  |
| --- | --- | --- | --- | --- | --- | --- |
| **Brain region** | **Neuropeptides**  **(pmol mg^‐1^)** | ***Sympatric cleaner*** | ***Allopatric cleaner*** | ***Conspecific*** | ***Ball*** | **Statistics** |
|  |  |  |  |  |  |  |
| **Forebrain** | **AVT** | 0.09±0.02 | 0.20±0.03 | 0.10±0.03 | 0.10±0.03 | **F_(3,35)_=3.28; *p=*0.03; R^2^=0.15** |
|  | **IT** | 0.15±0.03 | 0.16±0.04 | 0.16±0.03 | 0.17±0.03 | **F_(3,36)_=0.08; *p=*0.97; R^2^=-0.08** |
| **Diencephalon** | **AVT** | 0.13±0.04 | 0.18±0.04 | 0.14±0.03 | 0.19±0.05 | **F_(3,35)_=0.37; *p*=0.78; R^2^=-0.05** |
|  | **IT** | 0.15±0.04 | 0.34±0.05 | 0.17±0.04 | 0.20±0.04 | **F_(3,36)_=3.80; *p=*0.02; R^2^=0.18** |
| **Optic Tectum** | **AVT** | 0.07±0.01 | 0.12±0.05 | 0.07±0.01 | 0.07±0.02 | **F_(3,35)_=3.27; *p=*0.03; R^2^=0.15** |
|  | **IT** | 0.12±0.02 | 0.12±0.02 | 0.11±0.02 | 0.11±0.02 | **F_(3,36)_=0.22; *p=*0.88; R^2^=-0.06** |
| **Cerebellum** | **AVT** | 0.11±0.02 | 0.26±0.04 | 0.25±0.05 | 0.28±0.04 | **F_(3,35)_=4.82; *p=*0.007; R^2^=0.23** |
|  | **IT** | 0.21±0.03 | 0.22±0.04 | 0.21±0.03 | 0.18±0.03 | **F_(3,36)_=0.16; *p=*0.92; R^2^=-0.07** |
| **Brain Stem** | **AVT** | 0.22±0.04 | 0.28±0.04 | 0.20±0.04 | 0.25±0.06 | **F_(3,35)_=0.63; *p=*0.60; R^2^=-0.03** |
|  | **IT** | 0.23±0.05 | 0.26±0.05 | 0.24±0.05 | 0.28±0.05 | **F_(3,36)_=0.10; *p=*0.96; R^2^=-0.07** |

**Table C.** Tukey post-hoc HSD tests for arginine vasotocin (AVT) levels in different brain macro-areas across four experimental treatments. Significant tests are marked with * for p < 0.05.

|  | **Experimental** |  |  |  |  |
| --- | --- | --- | --- | --- | --- |
| **Brain regions** | **treatments** | ***Sympatric cleaner*** | ***Allopatric cleaner*** | ***Conspecific*** | ***Ball*** |
| **Forebrain** | ***Sympatric cleaner*** | ---------- | p=0.027* | p=0.986 | p=1.000 |
|  | ***Allopatric cleaner*** | p=0.027* | ---------- | p=0.065 | p=0.035* |
|  | ***Conspecific*** | p=0.986 | p=0.065 | ---------- | p=0.992 |
|  | ***Ball*** | p=1.000 | p=0.035* | p=0.992 | ---------- |
|  |  |  |  |  |  |
|  |  |  |  |  |  |
| **Optic Tectum** | ***Sympatric cleaner*** | ---------- | p=0.041* | p=1.000 | p=0.993 |
|  | ***Allopatric cleaner*** | p=0.041* | ---------- | p=0.051 | p=0.081 |
|  | ***Conspecific*** | p=1.000 | p=0.051 | ---------- | p=0.996 |
|  | ***Ball*** | p=0.993 | p=0.081 | p=0.996 | ---------- |
|  |  |  |  |  |  |
| **Cerebellum** | ***Sympatric cleaner*** | ---------- | p=0.067 | p=0.070 | p=0.012* |
|  | ***Allopatric cleaner*** | p=0.067 | ---------- | p=0.998 | p=0.958 |
|  | ***Conspecific*** | p=0.070 | p=0,998 | ---------- | p=0.892 |
|  | ***Ball*** | p=0.012* | p=0.958 | p=0.892 | ---------- |
|  |  |  |  |  |  |

**Table D.** Tukey post-hoc HSD tests for isotocin (IT) levels in Diencephalon across four experimental treatments. Significant tests are marked with * for p < 0.05.

|  | **Experimental** |  |  |  |  |
| --- | --- | --- | --- | --- | --- |
| **Brain region** | **treatments** | ***Sympatric cleaner*** | ***Allopatric cleaner*** | ***Conspecific*** | ***Ball*** |
|  |  |  |  |  |  |
| **Diencephalon** | ***Sympatric cleaner*** | ---------- | p=0.016***** | p=0,983 | p=0,827 |
|  | ***Allopatric cleaner*** | p=0.016***** | ---------- | p=0.043***** | p=0.099 |
|  | ***Conspecific*** | p=0.983 | p=0.043***** | ---------- | p=0.965 |
|  | ***Ball*** | p=0.827 | p=0.099 | p=0.965 | ---------- |
|  |  |  |  |  |  |

**Table E.** Correlations (Pearson correlation coefficients) between each behavioural measure and brain arginine vasotocin (AVT) levels in different brain macro-areas: forebrain, diencephalon, optic tectum, cerebellum and brain stem; for two experimental treatments: clients introduced to sympatric cleaners and clients introduced to conspecifics. Significant correlations are highlighted in bold marked with * for p < 0.05. However, no correlations were significant after calculation of Hochberg-adjusted p-values^2^

| Behaviour | Brain Macro-areas | | | | |
| --- | --- | --- | --- | --- | --- |
|  | Forebrain | Diencephalon | Optic Tectum | Cerebellum | Brain  Stem |
| Sympatric cleaners  N=10 |  |  |  |  |  |
| Frequency of cleaning interactions | **r = - 0.64**,^*^  **P = 0.04** | r = - 0.29,  P = 0.41 | r = - 0.43,  P = 0.22 | r = - 0.28,  P = 0.43 | r = - 0.34,  P = 0.34 |
| Inspection duration | r = - 0.41,  P = 0.24 | r = - 0.30,  P = 0.39 | r = - 0.35,  P = 0.32 | **r = - 0.66, ^*^**  **P = 0.04** | r = - 0.18,  P = 0.62 |
| Proportion of interactions with tactile stimulation | r = 0.11,  P = 0.77 | r = 0.13,  P = 0.72 | r = 0.29,  P = 0.41 | r = 0.04,  P = 0.92 | r = - 0.22,  P = 0.53 |
| Proportion of time providing tactile stimulation | r = - 0.14,  P = 0.69 | r = - 0.19,  P = 0.59 | r = - 0.05,  P = 0.89 | r = - 0.15,  P = 0.69 | r = - 0.45,  P = 0.19 |
| Jolts (per 100s of inspection) | r = - 0.48,  P = 0.16 | r = - 0.13,  P = 0.71 | r = - 0.20,  P = 0.57 | r = - 0.26,  P = 0.48 | r = - 0.01,  P = 0.98 |
| Frequency of chases (by focal) | r = - 0.53,  P = 0.12 | r = - 0.26,  P = 0.47 | r = - 0.43,  P = 0.22 | r = - 0.22,  P = 0.54 | r = 0.03,  P = 0.93 |
| Chase duration  (in s) | r = - 0.52,  P = 0.12 | r = - 0.26,  P = 0.46 | r = - 0.41,  P = 0.24 | r = - 0.19,  P = 0.59 | r = 0.04,  P = 0.92 |
| Conspecifics  N=9 |  |  |  |  |  |
| Frequency of chases (by focal) | r = - 0.21,  P = 0.59 | r = - 0.46,  P = 0.22 | r = - 0.44,  P = 0.24 | r = - 0.64,  P = 0.06 | r = - 0.29,  P = 0.45 |
| Chase duration  (in s) | r = - 0.30,  P = 0.45 | r = - 0.59,  P = 0.09 | r = - 0.39,  P = 0.29 | **r = - 0.71,^*^**  **P = 0.03** | r = - 0.48,  P = 0.19 |
| Frequency of bites (provided by focal) | r = - 0.10,  P = 0.79 | r = - 0.22,  P = 0.57 | r = - 0.47,  P = 0.20 | r = - 0.07,  P = 0.83 | r = - 0.05,  P = 0.88 |

**Table F.** Correlations (Pearson correlation coefficients) between each behavioural measure and brain isotocin (IT) levels in different brain macro-areas: forebrain, diencephalon, optic tectum, cerebellum and brain stem; for two experimental treatments: clients introduced to sympatric cleaners and clients introduced to conspecifics.

| Behaviour | Brain Macro-areas | | | | |
| --- | --- | --- | --- | --- | --- |
|  | Forebrain | Diencephalon | Optic tectum | Cerebellum | Brain stem |
| Sympatric cleaners  N=11 |  |  |  |  |  |
| Frequency of cleaning interactions | r = 0.49  P = 0.13 | r = - 0.10  P = 0.77 | r = - 0.59  P = 0.06 | r = - 0.06  P = 0.85 | r = - 0.07  P = 0.84 |
| Inspection duration | r = - 0.43  P = 0.18 | r = 0.05  P = 0.89 | r = - 0.28  P = 0.40 | r = - 0.29  P = 0.38 | r = - 0.29  P = 0.38 |
| Proportion of interactions with tactile stimulation | r = 0.04  P = 0.91 | r = - 0.51  P = 0.11 | r = 0.08  P = 0.82 | r = -0.53  P = 0.09 | r = 0.03  P = 0.93 |
| Proportion of time providing tactile stimulation | r = - 0,14  P = 0.69 | r = - 0.54  P = 0.09 | r = - 0.13  P = 0.70 | r = - 0.59  P = 0.05 | r = - 0.01  P = 0.97 |
| Jolts (per 100s of inspection) | r = - 0.51  P = 0.11 | r = - 0.07  P = 0.84 | r = - 0.49  P = 0.13 | r = 0.20  P = 0.55 | r = 0.16  P = 0.65 |
| Frequency of chases (by focal) | r = - 0.56  P = 0.07 | r = - 0.26  P = 0.45 | r = - 0.44  P = 0.17 | r = 0.001  P = 0.999 | r = - 0.05  P = 0.88 |
| Chase duration  (in s) | r = -0.55  P = 0.08 | r = -0.11  P = 0.76 | r = -0.42  P = 0.20 | r = -0.04  P = 0.92 | r = -0.11  P = 0.74 |
|  |  |  |  |  |  |
| Conspecifics  N=10 |  |  |  |  |  |
| Frequency of chases (by focal) | r = - 0.47  P = 0.17 | r = - 0.34  P = 0.34 | r = - 0.38  P = 0.28 | r = - 0.32  P = 0.37 | r = - 0.31  P = 0.40 |
| Chase duration  (in s) | r = - 0.62  P = 0.06 | r = - 0.50  P = 0.15 | r = - 0.56  P = 0.09 | r = - 0.54  P = 0.11 | r = - 0.51  P = 0.14 |
| Frequency of bites (provided by focal) | r = 0.30  P = 0.39 | r = 0.18  P = 0.61 | r = 0.31  P = 0.39 | r = 0.55  P = 0.10 | r = 0.11  P = 0.77 |

**Fig. A.**

i)

ii)

Figure Caption

**Fig A.** **Calibration curves for AVT (i) and IT (ii).** Peak area vs. concentration of standard peptide (pmol/ml). Data are given in Table A.

References

1. Hochberg Y. A Sharper Bonferroni Procedure for Multiple Significance Testing. Biometrika. 1988: 75; 800–803.

2. Mori Y, Kano Y. Changes in plasma concentration of LH, progesterone and estradiol in relation to the occurrence of luteolysis, oestrus and time of ovulation in the Shiba goat (*Capra hircus*). J Reprod Fertil. 1984: 72**;** 223- 230.
